# Quantifying interplay effects in lung cancer IMPT: a comprehensive analysis of treatment planning parameter sensitivity

**DOI:** 10.1186/s12885-025-15416-x

**Published:** 2025-12-05

**Authors:** Xiaoying Fan, Shuting Wang, Weijie Li, Ruozheng Wang, Yong Yin, Tianyuan Dai

**Affiliations:** 1 Department of Graduate, Shandong First Medical University, Shandong Academy of Medical Sciences, Jinan, China; 2 Department of Radiation Oncology Physics and Technology, Shandong Cancer Hospital and Institute, Shandong First Medical University and Shandong Academy of Medical Sciences, Jinan, China; 3 College of Intelligence and Information Engineering, Shandong University of Traditional Chinese Medicine, Jinan, China; 4 Affilated Cancer Hospital, The Third Affillated Teaching Hospital of Xinjiang Medical University, Urumqi, China

**Keywords:** Lung cancer, 4D dynamic dose, Treatment planning parameter sensitivity, Intensity modulated proton therapy, interplay effect

## Abstract

**Purpose:**

Precise dose delivery to the target is constrained by respiratory motion in intensity modulated proton therapy (IMPT) for lung cancer. This study aims to investigate the impact of treatment planning parameters on the lung cancer IMPT.

**Materials and methods:**

30 lung cancer patients treated at Shandong Cancer Hospital and institute were recruited in the study. A 4DCT dataset with 10 respiratory phases was reconstructed for each patient. The RayStation11B treatment planning system was used to create two-oblique-field IMPT plans. A prescription dose of 60 Gy (RBE) for 30 fractions was administered to 95% volume of the CTV. Five proton planning strategies were developed for each patient: (1) Range shifter (RS) plan: four plans were generated by varying range shifter thickness (0, 2, 3, and 5 cm) to change spot sizes; (2) Layer Spacing (LS) plan: four plans with different layer spacing (0.5, 1, 1.5 and 2 cm); (3) Spot Spacing (SS) plan: four plans with spot spacing variations (0.5, 1, 1.5 and 2 cm); (4) Repainting (RE) plan: layer repainting techniques with different numbers of repainting 1, 4 or 8 was adopted to the plan; (5) Ring plan: Dose fall-off was optimized using a ring structure around the target. 4D dynamic dose distributions (4DDDs) were calculated for plans created with various treatment planning parameters. Plan quality and dose-volume histogram (DVH) parameters for the target and organs at risk (OARs) were then analyzed.

**Results:**

For the RS plan, the D_95%_ of CTV, V_20_ and V_5_ of ipsilateral lung increased as the spot size became larger. The D_2%_ in RS5 plan was slightly higher than in the other three groups. The D_95%_ and D_2%_ in the target decreased as the layer spacing increased. The average value for the ELS2 group was about 3.8 Gy (RBE) higher than that of the ELS0.5 group (69.7 vs. 65.9 Gy (RBE)). The values for V_20_ and V_5_ of lung were similar across groups. Variations in spot spacing had slightly impact on D_95%_ for the target and V_20_, V_5_ for the ipsilateral lung. The effect on D_2%_ was more pronounced. The median D_95%_ values for the three groups gradually increased with the number of repainting. For the ipsilateral lung, the number of repainting had little impact on the doses of V_20_ and V_5_. For the ring plan, the nominal group had slightly higher D_95%_, V_20_, and V_5_ values compared to the ring group. The D_2%_ in the nominal group was lower than that in the ring group, with average values of 65.8 and 67.6 Gy (RBE), respectively.

**Conclusion:**

A systematic analysis was conducted in this study on the impact of spot size, LS, SS, layer repainting, and ring structure on the 4D robustness of IMPT plans for lung cancer patients. These five strategies demonstrated that beam spot size and layer spacing affected plan robustness, while spot spacing had relatively minor influence on robustness. The repainting technology can mitigate the interplay effect caused by respiratory motion. The application of ring structures affected the dose distribution of the moving target.

## Introduction

Lung cancer remains one of the most prevalent malignancies globally in terms of both incidence and mortality [1, 2]. Radiotherapy plays a pivotal role in treating early-stage inoperable or locally advanced lung cancer. However, conventional photon therapy may cause excessive dose exposure to organs at risk (OARs), such as the heart and spinal cord, due to limitations in physical dose deposition.

Proton therapy, leveraging the unique Bragg peak characteristics, achieves sharp dose fall-off at the distal beam range. Consequently, radiation exposure to normal tissues is significantly reduced [3, 4]. Recently, pencil beam scanning (PBS) has emerged as the preferred delivery method for proton therapy due to its well dose conformity [5]. However, proton therapy is more susceptible to CT density variations, patient setup uncertainties and tumor motion [6]. Respiratory motion of lung tumors and PBS delivery may cause interplay effect. Uncertainties in tumor motion may compromise dose delivery to target, potentially causing insufficient local coverage or excessive doses to critical organs. These effects could ultimately impair tumor control and increase radiation-induced complication risks [7].

To mitigate the effects, clinical motion management strategies such as breath-hold techniques and respiratory gating are commonly utilized [8]. While these methods reduce motion amplitudes, they may compromise patient comfort or prolong treatment time [9]. These factors could decrease patient compliance. Consequently, target dose coverage may be compromised. 4D robust optimization can also alleviate interplay effects [10]. However, 4D robust optimization requires consideration of multiple motion scenarios, resulting in significantly longer computation times and reduced clinical efficiency. Furthermore, 4D optimization has not been widely adopted in routine practice. Similarly, studies have demonstrated that 3D robust optimization effectively addresses uncertainties from CT density variations and setup uncertainties, particularly for tumors with limited motion [11, 12]. Nevertheless, for lung tumors exhibiting large respiratory motion amplitudes, interplay effect remains pronounced. Therefore, meticulous parameter selection and motion mitigation strategies must be carefully implemented in IMPT treatment planning for such cases [13].

This study aims to investigate the impact of treatment planning parameter variations on dose distribution in lung cancer IMPT. For this study, three parameters: spot size, energy layer spacing (ELS) and spot spacing (SS) were adjusted during the IMPT treatment planning to explore their effects on interplay effects. Furthermore, ring structure was incorporated during plan optimization to study how ring constrains affect the interplay effect. Energy layer repainting technology was investigated to evaluate the mitigation of interplay effects with different numbers of repainting (1, 4, and 8). A script was utilized to calculate 4D dynamic dose (4DDD) by considering the interplay effect of dynamic proton beam delivery and respiratory motion. Dose-volume histogram (DVH) parameters of these strategies were used to quantitatively evaluate the interplay effects.

## Materials and methods

This study aimed to evaluate the impact of planning parameter variations on the interplay effect in IMPT for lung cancer. Five parameters were selected for the sensitivity analysis. Spot size: proton pencil beam spot size was adjusted by utilizing various range shifters(RS) with different thickness. Energy layer spacing (ELS): Defined as the depth interval between adjacent Bragg peaks of energy layers. It determines the longitudinal sampling resolution, influencing dose homogeneity along the depth and delivery efficiency. Spot spacing (SS): Defined as the spatial distance between adjacent scanning spots in each energy layer. It controls the lateral sampling density, affecting target dose smoothness and motion sensitivity. Repainting technology: Applies repeated layer scanning to mitigate motion-induced interplay effects and enhance the temporal and spatial robustness of dose delivery. Ring structure: introduced around the target to create a dose fall-off zone, reducing high-dose spill and improving spatial dose distribution. The parameter ranges were defined based on the parameter availability and clinical practice at our institution, the adjustable limits of the RayStation treatment planning system, and commonly adopted settings reported in previous studies [14–17].

### Patients and contours

30 lung cancer patients treated at Shandong Cancer Hospital and institute were included in the study. All CTs were acquired under free-breathing using a Philips Brilliance Big Bore CT scanner (Philips, Netherlands). A 4DCT dataset with 10 respiratory phases was reconstructed for each patient. CT scans were acquired in head-first supine position (3 mm slice thickness). On each 4DCT dataset, Gross tumor volume (GTV) and organs at risk (OARs) were manually defined by radiation oncologists using the RayStation 11B treatment planning system. The clinical target volume (CTV) was generated by uniformly expanding the GTV by 8 mm [18, 19]. The CTV ranged from 13.48 to 259.28 cc (median 41.55 cc). We measured the centroid coordinates (x, y, z axes) of CTVs across all 10 respiratory phases in the 4DCT datasets. The tumor motion amplitude was defined as the maximum displacement in three coordinate directions among all phases [20]. CTV motion amplitudes ranged from 0.64 to 11.82 mm (median 5.03 mm). Table 1 summarizes the corresponding patients, tumor location, volume and motion amplitude.

**Table 1:** Patient number, tumor volume, tumor location and motion amplitude in this study

Patient number	GTV (cc)	CTV (cc)	Tumor location	Motion amplitude (mm)
1	9.25	41.15	Right lung	1.54
2	16.43	94.16	Left lung	6.87
3	37.82	116.34	Right lung	4.07
4	7.58	40.67	Right lung	4.00
5	65.46	186.86	Left lung	5.54
6	6.10	33.05	Left lung	5.35
7	4.72	27.61	Right lung	4.01
8	21.45	73.6	Right lung	4.71
9	9.49	44.35	Right lung	3.62
10	92.41	231.33	Left lung	2.38
11	38.04	119.9	Right lung	6.42
12	9.23	41.79	Right lung	7.44
13	28.64	84.86	Left lung	0.64
14	36.96	107.67	Right lung	8.70
15	11.46	52.38	Left lung	9.13
16	110.97	259.28	Left lung	4.16
17	23.97	82.88	Left lung	6.98
18	6.72	33.91	Left lung	9.56
19	7.21	37.29	Left lung	11.82
20	12.56	57.79	Right lung	5.60
21	4.29	30.43	Right lung	3.19
22	9.55	41.31	Right lung	1.89
23	4.53	35.76	Right lung	3.96
24	5.96	34.4	Left lung	7.83
25	6.20	36.76	Left lung	6.07
26	2.79	23.23	Right lung	1.92
27	1.13	13.48	Right lung	7.96
28	7.01	39.22	Left lung	2.67
29	4.19	29.31	Right lung	8.16
30	12.81	54.25	Left lung	1.68

### Treatment planning

At the Proton Center of Shandong Cancer Hospital, the ProBeam clinical beam model (Varian Medical Systems, CA, USA) was employed. The beam energies ranged from 70 to 240 MeV in spot scanning mode. A Monte Carlo algorithm was used to calculate the dose with a 3 × 3 × 3 mm^3^ grid [21]. The end-exhalation phase (denoted as 0%Ex) from the 4DCT dataset served as the reference phase in this study [10]. The RayStation11B treatment planning system was used to create two-oblique-field IMPT plans. A prescription dose of 60 Gy (RBE) for 30 fractions was administered to 95% volume of the CTV [22]. The relative biologic effectiveness (RBE) weighted dose was determined using a fixed RBE value of 1.1 for proton.

Five proton planning strategies were simulated for each patient: (1) Range shifter (RS) plan: increasing range shifter thickness resulted in larger spot sizes [14]. Four plans were generated by varying range shifter thickness (0, 2, 3, and 5 cm) to modulate different spot sizes. These were labeled as RS0, RS2, RS3, RS5 groups. (2) Layer Spacing (LS) plan: IMPT plans with different layer spacing (0.5, 1, 1.5 and 2 cm) were modulated, designated as ELS0.5, ELS1, ELS1.5, ELS2 groups. (3) Spot Spacing (SS) plan: spot spacing variations (0.5, 1, 1.5 and 2 cm) were modulated, designated as SS0.5, SS1, SS1.5, SS2 groups. (4) Repainting (RE) plan: layer repainting techniques with different numbers of repainting 1, 4 and 8 was adopted to the IMPT plans, designated as RE1, RE4, RE8 groups. (5) Ring plan: Dose fall-off was optimized using a ring structure (Boolean of CTV + 1 cm/3 cm expansions). Ring structure was adopted to plans, designated as ring group. The non-ring nominal plan was designated as nominal group.

In the RayStation treatment planning system, the default layer spacing (ELS) and spot spacing (SS) are automatically calculated based on the proton energy of each layer [16]. ELS and SS can be an automatic with scale. Variable distance depending on the Bragg peak width (energy) for ELS and projected sigma for SS. In our study, some plans are identical because only one parameter was modified at a time while the others were kept at their default settings.

### Planning optimization

The robust optimization functions were set as follows: The minimum dose of CTV was 60 Gy(RBE) with the weight of 100. The maximum dose of CTV was 62 Gy(RBE) with the weight of 100. The dose limits for OARs were as follows: the ipsilateral lung V_20_ < 35% with the weight of 40 (V_20_ defined as the normalized volume of ipsilateral lung receiving 20 Gy(RBE)), V_5_ < 65% with the weight of 40 [22]. Maximum spinal cord dose (D_max_) < 45 Gy(RBE) with the weight of 40. A maximum dose constraint was assigned to the ring structure to limit dose spill. The maximum dose of ring < 80% of prescription dose with the weight of 40. Among these five strategies, all other optimization parameters remained identical. The RS0, ELS1, SS1, RE1, and nominal groups shared identical parameter configurations.

### Dose calculation

**Fig. 1:**
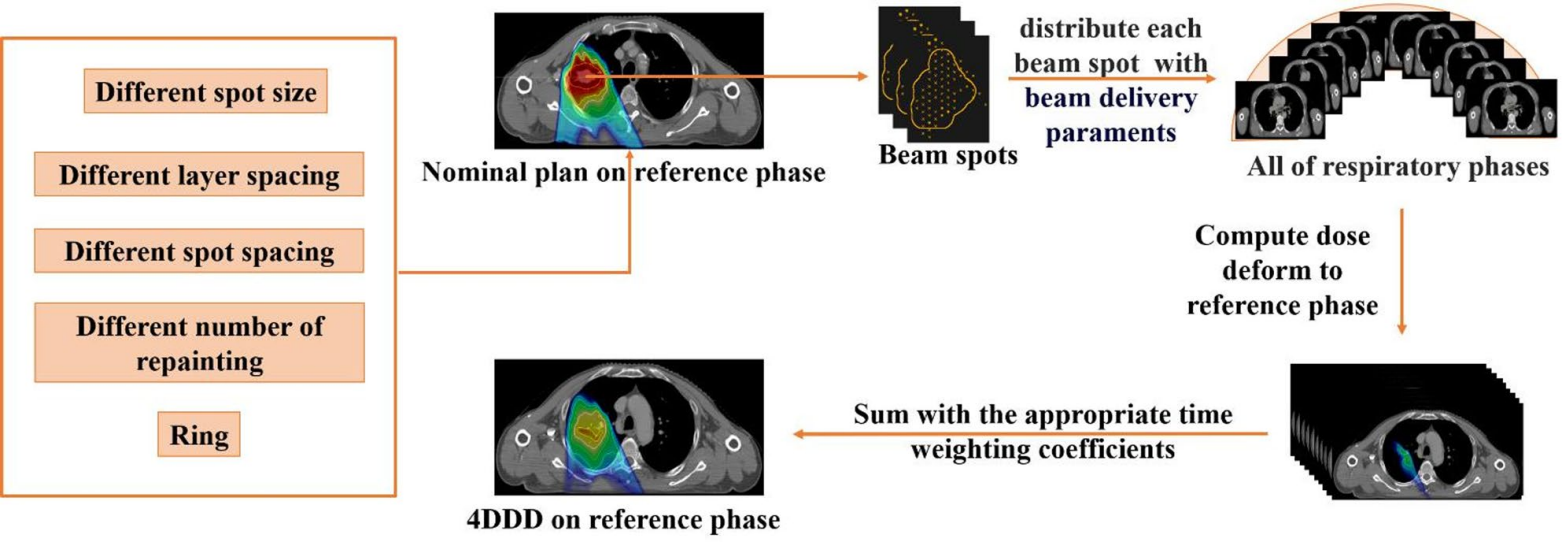
Workflow of 4DDD calculation in different strategies

This study investigated the interplay effect caused by dynamic pencil beam delivery and respiratory motion. A proton therapy simulation script [23] was developed in RayStation using the Monte Carlo dose algorithm to calculate the 4D dose distribution (4DDD). The script treatment parameters include: (1) The energy layer switching time was 1 s; (2) The beam spot speed was 10 m/s; (3) The beam dose rate was 50,000 clinical-MU/min (approximately 800 MU/s). Total duration of respiratory cycle was assumed to be 5 s. For RE plans, a special parameter “once per MU” was applied, determined by the spot MU in each plan. The breathing pattern was represented as a sin curve. All phases were equally distributed in time throughout the respiratory cycle. A scheme for the process of these two types of dose calculation was shown in Fig. 1.

### Dose evaluation

The nominal plans were normalized with 100% prescription dose covering 95% CTV. All five strategies used the same DVH parameters for evaluation: D_95%_ (prescription dose received by at least 95% of volume) and D_2%_, ipsilateral lung V_20_ and V_5_. The 4DDD of different parameter plans were compared to analyze interplay effects. Linear fitting was applied to regular CTV D_95%_ parameters. For each strategy, the minimal parameter configuration served as the reference (nominal group as the reference for the ring plan). All statistical analyses were performed in SPSS. Differences in dosimetric parameters were assessed using the Wilcoxon rank-sum test, with *p* < 0.05 considered significant.

## Results

### Spot size/range shifter

**Fig. 2:**
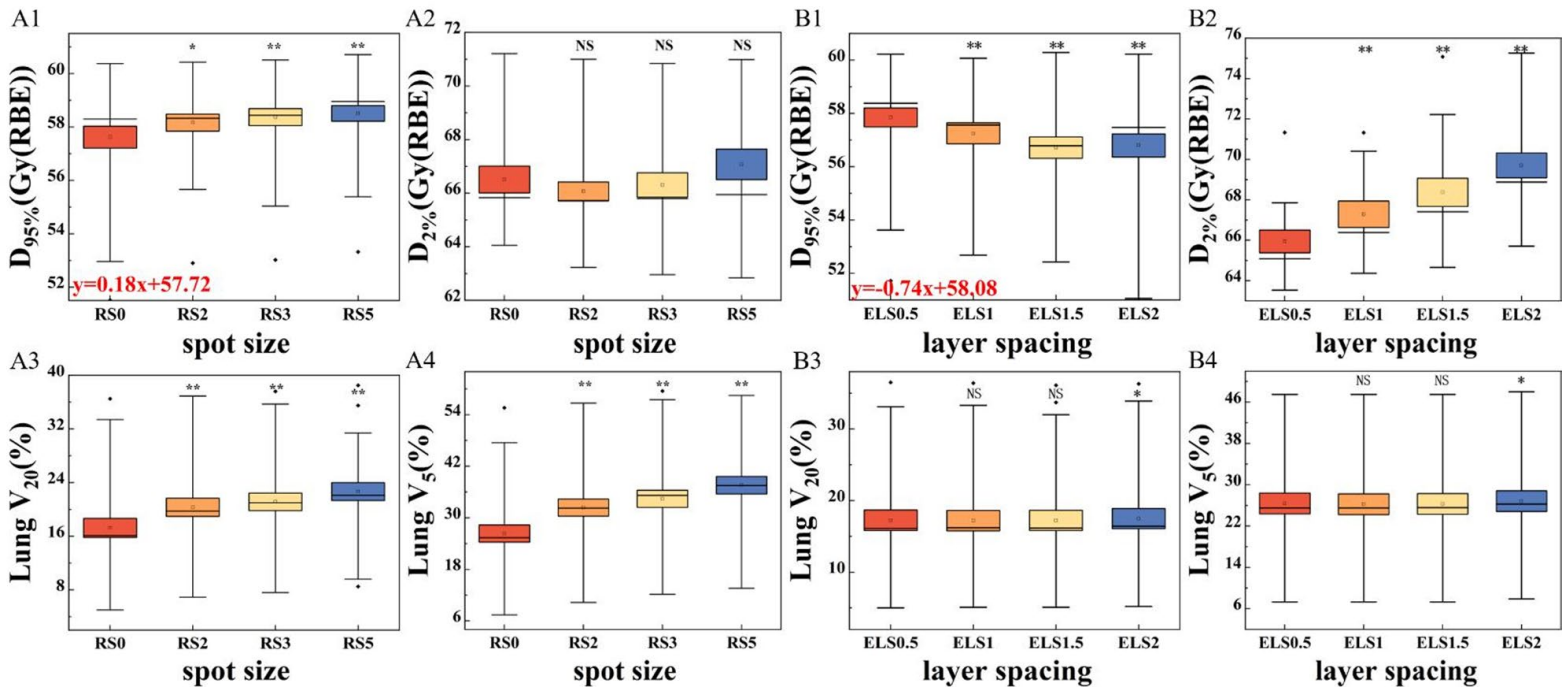
Panel A of the boxplots present the DVH parameters for the RS plan, panel B shows those for the LS plan. The DVH parameters include the D_95%_ (1) and D_2%_ (2) for CTV, V_20_ (3) and V_5_ (4) for ipsilateral lung. The differently colored boxes represent plans optimized with various parameters. Boxes represent interquartile range (IQR, 25th–75th percentiles), horizontal lines inside boxes indicate medians, and whiskers extend to 1.5×IQR. Outliers (individual dark points) are defined as values beyond 1.5×IQR from the box edges. The red formula in the figure represents the linear fitting result for this DVH parameter. The symbols above the dots are the results referring to the statistical significance analysis of the differences of the dose metrics (** *p* ≤ 0.001; * *p* ≤ 0.05; NS, *p*>0.05)

**Fig. 3:**
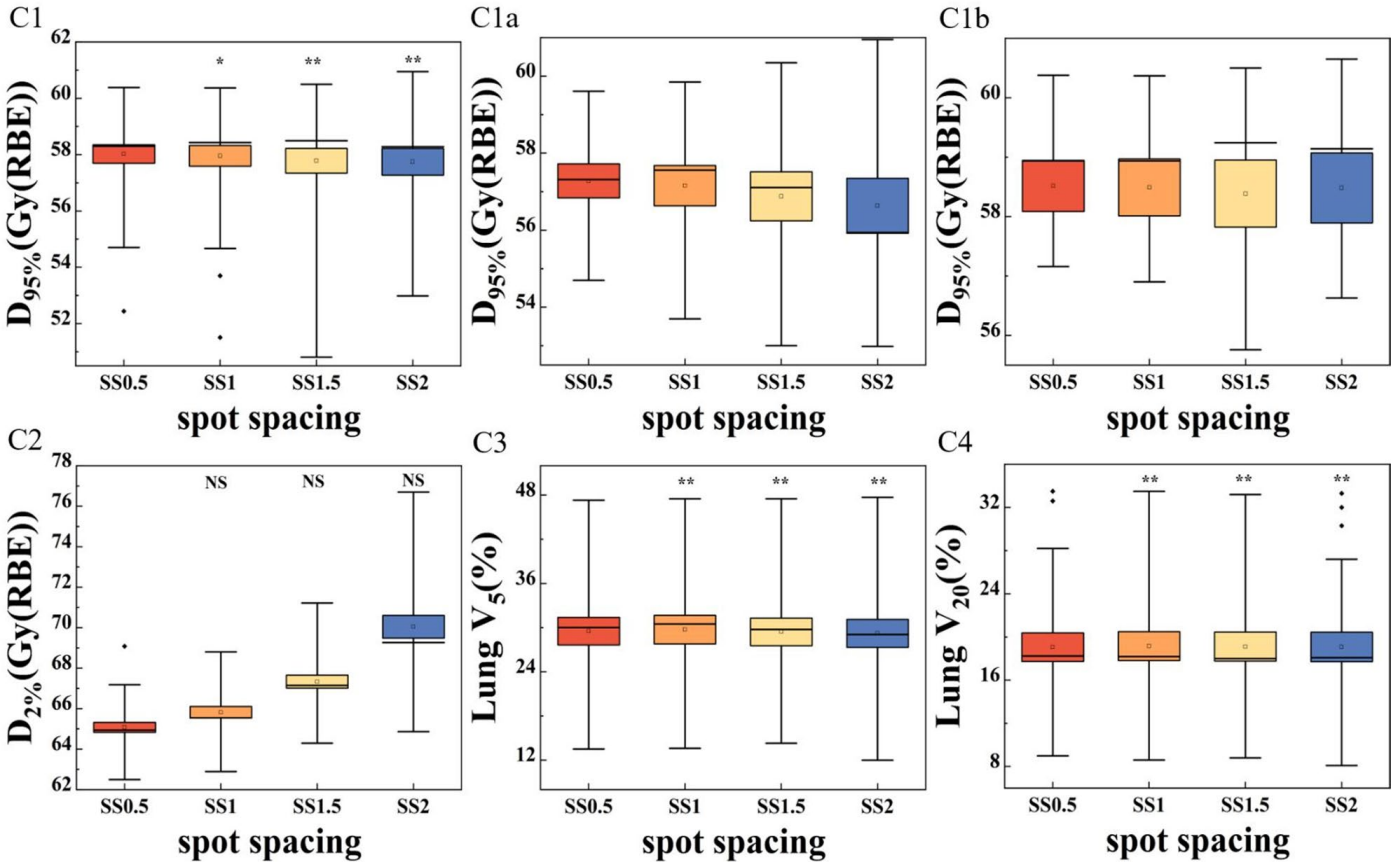
The boxplots present the D_95%_ (C1) and D_2%_ (C2) for CTV, V_20_ (C3) and V_5_ (C4) for ipsilateral lung for the SS plan. Panel C1a presents the D_95%_ for patients with the target motion amplitude greater than 6 mm, while Panel C1b shows the D_95%_ for patients with a target motion amplitude less than 6 mm. The differently colored boxes represent plans optimized with various spot spacing. Boxes represent interquartile range (IQR, 25th–75th percentiles), horizontal lines inside boxes indicate medians, and whiskers extend to 1.5×IQR. Outliers (individual dark points) are defined as values beyond 1.5×IQR from the box edges. The symbols above the dots are the results referring to the statistical significance analysis of the differences of the dose metrics (** *p* ≤ 0.001; * *p* ≤ 0.05; NS, *p*>0.05)

As shown in Fig. 2A, for the RS plan, the D_95%_ of CTV (A1) increased as the spot size became larger. The median D_95%_ was the highest for RS5 plan, which utilize the largest spot size. The RS0 plan, which had the smallest spot size (*p* < 0.05), resulted in the lowest D_95%_ for CTV. The D_2%_ value in RS5 (A2) was slightly higher than these in other three groups. The difference was not statistically significant (*p* > 0.05). For the ipsilateral lung, V_20_ (A3) and V_5_ (A4) increased with increasing spot size. That is to say that, as the spot size increased, the irradiated lung volume also increased. This dose increase was statistically significant (*p* ≤ 0.001). The dose distribution for a representative case was shown on the panel spot size of Fig. 3. Dose coverage gradually improved from A (RS0 group) to D (RS5 group).

### Layer spacing

For the LS plan (Fig. 2B), the D_95%_ of the target decreased as the layer spacing increased. The ELS0.5 group had the best dose coverage. However, D_2%_ increased with increasing layer spacing. The average value of D_2%_ for the ELS2 group was about 3.8 Gy (RBE) higher than that of the ELS0.5 group (69.7 Gy (RBE) vs. 65.9 Gy (RBE)). Both the statistical differences in D_95%_ and D_2%_ were significant (*p* < 0.001). The values for V_20_ and V_5_ of lung were similar across groups. There was no statistical significance between ELS1 and ELS1.5. The dose distribution for a representative case was shown on the panel layer spacing of Fig. 3. Dose coverage decreased progressively while D_2%_ increased from A (ELS0.5 group) to D (ELS2 group).

### Spot spacing

Figure 3 presented the DVH parameters for the SS plan. Variations in spot spacing had slightly impact on D_95%_ for the target and V_20_, V_5_ for the ipsilateral lung. The differences were statistically significant. In contrast, the effect on D_2%_ was more pronounced. As spot spacing increased, the D_2%_ value gradually increased. These difference was not statistically significant. To investigate whether motion amplitude affects the dependence of D_95%_ for the target on spot spacing, panel C1a presented the D_95%_ for patients with the target motion amplitude greater than 6 mm, while Panel C1b showed the D_95%_ for patients with a target motion amplitude less than 6 mm. Panel C1a demonstrated a slight decrease in target dose coverage as spot spacing increased. Panel C1b showed that there was no obvious D_95%_ on spot spacing. The values of D_95%_ were comparable across the four plans for the patients with a target motion amplitude less than 6 mm. The dose distribution for a representative case was shown on the panel spot spacing of Fig. 3. Dose coverage slightly increased from A (SS0.5) to D (SS2), while hotspots rose markedly. 

### Repainting

**Fig. 4:**
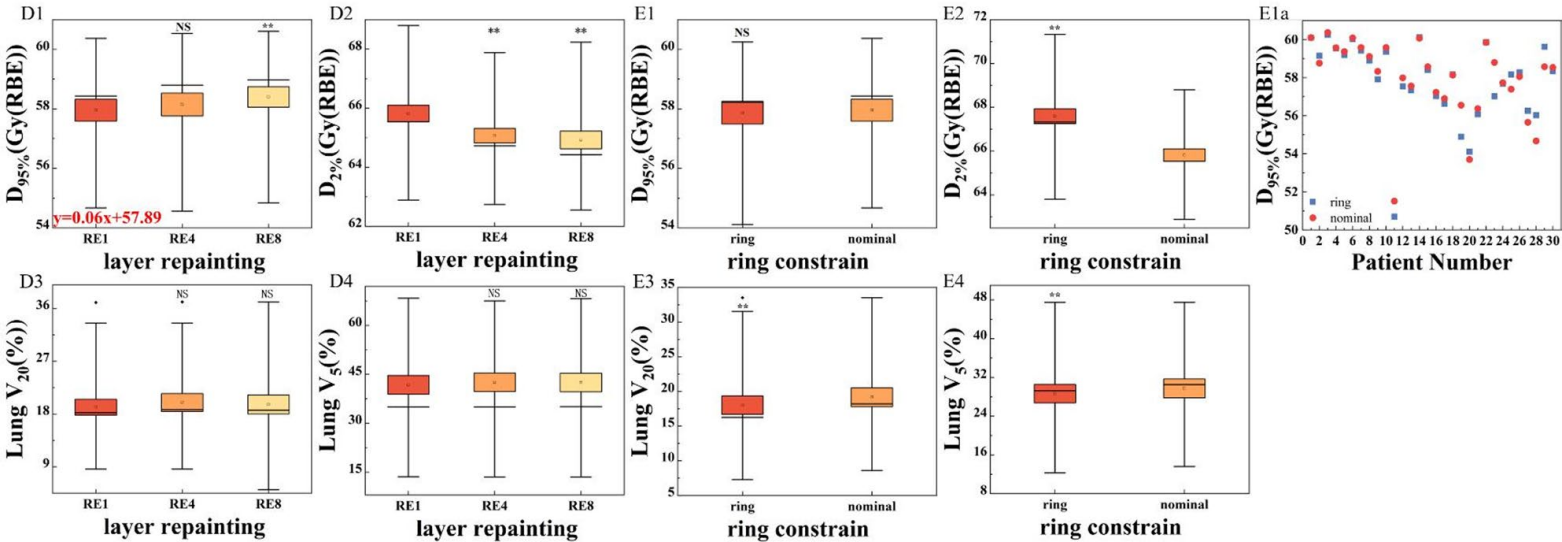
Panel D of the boxplots present the DVH parameters for the RE plan, panel E shows those for the ring plan. The DVH parameters include the D_95%_ (1) and D_2%_ (2) for CTV, V_20_ (3) and V_5_ (4) for ipsilateral lung. The differently colored boxes represent plans optimized with various parameters. Boxes represent interquartile range (IQR, 25th–75th percentiles), horizontal lines inside boxes indicate medians, and whiskers extend to 1.5×IQR. Outliers (individual dark points) are defined as values beyond 1.5×IQR from the box edges. The red formula in the figure represents the linear fitting result for this DVH parameter. The symbols above the dots are the results referring to the statistical significance analysis of the differences of the dose metrics (** *p* ≤ 0.001; * *p* ≤ 0.05; NS, *p*>0.05). Panel E1a displays the D_95%_ values of the target, arranged in ascending order of target motion amplitude for 30 patients. The red dots represent the 4DDD results for plans without ring contraction, while the blue squares indicate the 4DDD results for plans with ring contraction.

Figure 4D presented the D_95%_ (D1) and D_2%_ (D2) for CTV, V_20_ (D3) and V_5_ (D4) for ipsilateral lung for the RE plan. In panel D1, the median value of the D_95%_ for RE8 was higher than the other groups. The median D_95%_ values for the three groups gradually increased with the number of repainting. The trend in panel D_2_ was opposite to that in D1. These differences were statistically significant, except for the D_95%_ in RE4 plan. For the ipsilateral lung, the number of repainting had little impact on V_20_ and V_5_. There was no statistically significant difference for the dose of lung. The dose distribution for a representative case was shown on the panel layer repainting of Fig. 3. Dose coverage improved progressively from A (RE1 group) to C (RE8 group).

### Ring

In Fig. 4E, the D_95%_ (E1) and D_2%_ (E2) for CTV, V_20_ (E3) and V_5_ (E4) for ipsilateral lung for the ring plan were shown. For the ring plan, the nominal group had slightly higher D_95%_, V_20_, and V_5_ values compared to the ring group. However, the D_2%_ in the nominal group was lower than in the ring group, with average values of 65.8 and 67.6 Gy (RBE), respectively. These differences were statistically significant, except for D_95%_. Panel E1a displayed the D_95%_ values of the target, arranged in ascending order of target motion amplitude for all 30 patients to show degradation of coverage in larger-motion cases. After approximately the 20th patient, the results of the two plans showed a random distribution. For the first to the 19th patient, target dose coverage was generally better in the nominal plan. The motion amplitude of the 20th patient was 6.42 mm. As shown in panel ring contraction of Fig. 3, the ring plan (B) produced a more central dose concentration, a steeper gradient, and higher hotspots.

## Disscusion

This study systematically analyzed the impact of various treatment planning parameter settings on interplay effects in proton therapy for lung cancer. During plan design, spot size can be modulated through RS adjustments. The thicker the range shifter, the larger the spot size. The results in this work demonstrated that larger spot sizes improved target coverage but increased doses to OARs. Several previous studies have demonstrated that smaller spot sizes reduce radiation exposure to surrounding normal tissues, thereby decreasing complication probabilities [24, 25]. Quan et al. reported that smaller spot sizes better protect normal tissues without compromising target dose delivery for head and neck patients [25]. In this study, the average D_95%_, V_20_, and V_5_ values in the RS0 group were 58.2 Gy(RBE), 17.24%, and 26.32%, respectively. In the RS5 group, the corresponding values were 59.6 Gy(RBE), 22.65%, and 37.58%. While the target dose increased, the dose to the ipsilateral lung also rose significantly. Therefore, balancing the target coverage and normal tissue complication through spot size modulation is critical during IMPT for lung cancer.

**Fig. 5:**
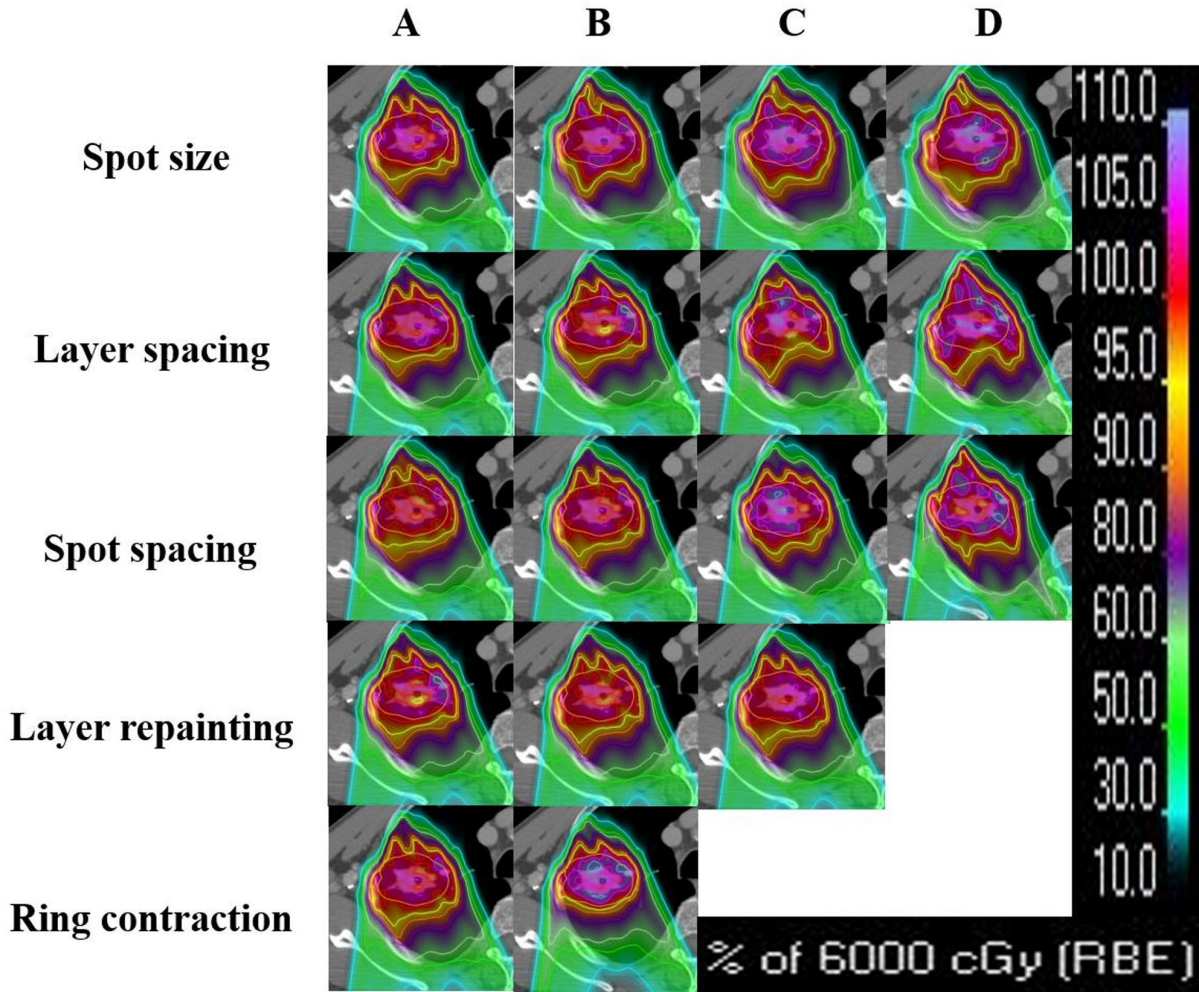
Dose distributions of a representative case for the five types of plans. The orange lines indicate the outlines of the CTVs. Panels A to D represent a strategy with parameter values increasing from small to large, such as from RS0 to RS5. In the panel of ring contraction, A represents the nominal plan and B represents the ring plan

During the creation of IMPT plans, proton spot locations and weights are first fixed in the therapy planning system. The dose distribution is then achieved by adjusting only the spot weighting during optimization [16]. Theoretically, smaller layer spacing and spot spacing result in more spots, providing greater flexibility during optimization. As a result, the optimized dose distribution was expected to be more conformal normally. In Fig. 5Ca-b, the 30 patients were divided into two groups based on tumor motion amplitude (using 6 mm as the threshold) to evaluate the D_95%_ of CTV. The results showed that smaller spot spacing slightly improved target dose for patients with larger motion amplitudes, as shown in Fig. 5C1a. For patients with smaller motion, as shown in Fig. 5C1b, the spot spacing effect on interplay effect was limited. Meanwhile, smaller spot spacing may help reduce the occurrence of hot spots and improve dose uniformity within the target, as shown in Fig. 5C2. However, it may also increase the complexity of the plan [26]. The mitigation of interplay effect using the small spot spacing for the situations with tumor motion amplitude less than 6 mm could be attributed to the more spots in the plan, acting as a repainting technique.

In contrast, layer switching requires more time(~ 1 s). More energy layers lead to more layer switch, which in turn increase the total beam on time. The results in this work demonstrated that the application of smaller layer spacing could mitigate interplay effect and increase the target coverage. However, Zhu et al. demonstrated that a smaller energy layer spacing increases the total beam delivery time [15]. Similarly, an increased number of spots also lead to longer delivery time. Prolonged beam delivery may cause respiratory fatigue, reduce patient comfort, and increase the risk of patient movement during treatment. These may pose challenges for clinical implementation on the settings of a smaller layer spacing in IMPT for lung cancer.

This study used a single ring structure configuration. The results showed that the ring structure affected the 4DDD of the target. In clinical applications, the width and boundary distance of the ring structure may vary depending on tumor size, location, and surrounding anatomy. The ring in this study was based on our institutional planning experience, which may limit the generalizability of the results. Future studies should explore different ring designs to evaluate their robustness under respiratory motion. As shown in Fig. 4D1, the use of iso-energy layer repainting significantly improved dose coverage in the target, especially when repeated eight times. Nevertheless, increasing the number of repainting also prolonged the actual beam delivery time [27], which may increase the likelihood of set up uncertainties. The optimal repainting number is still controversial because it varies with patient respiratory motion, energy switching time, and machine performance [28]. Therefore, its clinical application should be adjusted based on individual patient conditions.

In clinical practice, physicians are more likely to approve plans with dose distributions that are more conformal to the tumor target. The application of ring structures was common clinical practice in IMRT to create a dose fall-off around the target, which can protect the surrounding normal tissues better. However, proton beam delivery is more sensitive to respiratory motion and it is unknown that whether the application of ring structures would affect the 4D robustness of IMPT plan. The ring structure constrained dose spill outside the target, resulting in a steeper dose fall-off near the target boundary. This may explain the observations in Fig. 4E1, where the CTV 4DDD was lower than that of the nominal plan in some plans. In this study, this effect was more evident in patients with smaller motion amplitudes, as shown in Fig. 4E1a. For patients with larger motion, the strong interplay effect may have masked the dose differences caused by the ring structure. The impact of ring structures on patients with large tumor motion remains to be further investigated.

Although, previous studies have demonstrated that the applying IMPT to patients with motion amplitudes larger than 10 mm is challenge [18, 29] and should be carefully evaluated, this study still had some limitations. Among the 30 patients, only one patient showed tumor motion amplitude greater than 10 mm, which may limit the generalizability of the findings to patients with large motion amplitude of lung cancer patients. In addition, the parameter ranges chosen for the five strategies were relatively narrow, which may explain why some strategies did not show significant differences. The width of the ring structure also affects the dose distribution in the target. In this study, only the impact of the ring on intra-fractional motion was considered. Future studies could adjust the geometrical parameters and dose constrains of the ring structure to explore its effect on setup uncertainties during inter-fractional treatment.

## Conclusion

A systematic analysis was conducted in this study on the 4D robustness of spot size, ELS, SS, layer repainting, and ring structure on IMPT plans for lung cancer patients. These five strategies demonstrated that beam spot size and layer spacing affected plan robustness, while spot spacing had relatively minor influence on robustness. The repainting technology can mitigate the interplay effect caused by respiratory motion. The application of ring structures affected the dose distribution of the moving target. In clinical practice, these parameters should be elaborately adjusted according to patient characteristics to enhance plan quality.

## Data Availability

All data included in this study are available upon request by contact with the corresponding author.

## Abbreviations

IMPT: Intensity modulated proton therapy
4DDD: 4D dynamic dose
PBS: Pencil beam scanning
CT: Computed Tomography
GTV: Gross target volume
CTV: Clinical tumor volume
OARs: Organs at risk
MC: Monte Carlo
RBE: Relative biologic effectiveness
HFS: Head First Supine
DVH: Dose-volume histogram
PBT: Proton beam therapy
ELS: Energy layer spacing
SS: Spot spacing
RS: Range shifter
IQR: Interquartile range
